# Outcomes of the Mini-Open Technique for Achilles Tendon Repair: An Updated Systematic Review

**DOI:** 10.7759/cureus.81718

**Published:** 2025-04-04

**Authors:** Jimmy Wen, Mouhamad Shehabat, Daniel I Razick, Ihab Abed, Zohaer Muttalib, Vince Thomas, Isabel Taguinod, Denise Nadora, Megan Kou, Soham Kondle, Bruce Lehnert

**Affiliations:** 1 Physical Medicine and Rehabilitation, California Northstate University College of Medicine, Elk Grove, USA; 2 Surgery, California Northstate University College of Medicine, Elk Grove, USA; 3 Medicine, California Northstate University College of Medicine, Elk Grove, USA; 4 Internal Medicine, California Northstate University College of Medicine, Elk Grove, USA; 5 Emergency Medicine, California Northstate University College of Medicine, Elk Grove, USA; 6 Neurology, California Northstate University College of Medicine, Elk Grove, USA; 7 Orthopedic Surgery, California Northstate University College of Medicine, Elk Grove, USA; 8 Podiatry, SOAR Spine and Orthopedics, Redwood City, USA

**Keywords:** achilles tendon repair, achilles tendon rupture, aofas, atrs, mini-open approach

## Abstract

There is much debate regarding the optimal treatment for acute Achilles tendon rupture. Surgical repair consists of open repair, percutaneous repair, and mini-open repair. The purpose of this review is to evaluate studies reporting outcomes after mini-open Achilles tendon repair (ATR) through evaluation of return to activity (RTA), complication rates, and postoperative patient-reported outcomes (PROs). A systematic review search following the guidelines established by the Preferred Reporting Items for Systematic Reviews and Meta-Analyses was performed in three databases for studies including mini-open ATR. Study variables included title, author, publication date, study year, number of patients/Achilles, mean age, mean follow-up time, time to RTA/return to sport (RTS)/return to work (RTW), PROs, and rates of complications. Twenty-five studies, including 957 patients with a mean age of 39.34 ± 6.84 years and a mean follow-up time of 29.48 ± 17.64 months, were included in this study. The overall rates (range) of RTA (eight studies), RTS (12 studies), and RTW (eight studies) were 96.46% (82.3%-100%) at four months (3.8-7), 92.7% (82.3%-100%) at 5.4 months (4.3-6.1), and 100% at 2.2 months (0.6-4.5), respectively. The mean postoperative PROs of the American Orthopedic Foot and Ankle Score (11 studies), Achilles Tendon Total Rupture Score (eight studies), and Visual Analog Pain (four studies) were 95.5 (90.1-99.2), 90.3 (86-94.6), and 3.35 (0.2-8.85), respectively. The overall complication rate for mini-open repair was 8.05%, with the most common being sural nerve injury, rerupture, and skin adhesions. The mean rates of sural nerve injury and rerupture were 1.67% and 1.25%. Patients undergoing mini-open ATR demonstrated high rates of return to baseline activity, low rates of complications, and excellent postoperative PRO scores. Mini-open repair is a reliable technique with comparable functional outcomes to traditional open repair, with lower rates of infections and wound healing issues.

## Introduction and background

The Achilles tendon is the most commonly ruptured tendon, with a rate of 18 patients per 100,000 annually [1]. Traditionally, open surgical repair has been the most commonly used modality and is associated with improved postoperative functional outcomes, return to baseline activity rates, and lower rates of rerupture compared to nonoperative treatment [2-4]. However, open repair is associated with soft-tissue complications such as delayed wound healing, infections, nerve entrapment, and large scars [3,4]. Over the last decades, improved knowledge and equipment have led to the evolution of surgical treatment options.

Currently, there are two main surgical modalities for Achilles tendon rupture repair: open and minimally invasive (percutaneous, mini-open, and endoscopic-assisted) [3]. Minimally invasive surgeries (MIS) were developed to minimize soft tissue trauma, incision size, and soft tissue complications associated with open repair. The percutaneous approach was developed by Ma and Griffith in 1977 but was associated with high rates of sural nerve injury due to limited visualization of the tendon [5]. Mini-open repair is an intermediary between traditional open and percutaneous, aiming to combine the beneficial effects of both techniques. The goal of mini-open repair is to minimize soft-tissue damage and reduce the complications of open repair while maintaining similar outcomes [6]. The advantage over percutaneous repair is improved visualization through a small incision and using retractors, thus potentially reducing the risk of sural nerve injury [6]. Several recent studies have compared MIS and open repair and reported similar outcomes but reduced rates of complications such as infections and wound dehiscence [7,8].

This systematic review includes the most recent available literature. It aims to report on the outcomes for mini-open Achilles tendon repair (ATR), including analysis of rates of return to activity (RTA)/return to sport (RTS)/return to work (RTW), postoperative patient-reported outcomes (PROs), and the rates of complications such as sural nerve injury and reruptures. We hypothesize that mini-open repair will show favorable outcomes with lower rates of complications.

## Review

Methods

Search Strategy

A search following guidelines established by the Preferred Reporting Items for Systematic Reviews and Meta-analyses was performed in three databases on August 26, 2024: PubMed, Embase, and Google Scholar. The following keywords were used to perform the literature search: “mini-open”, “Achilles”, “outcomes”, “follow-up”, and “complication”.

A Patient, Intervention, Comparison, Outcome, Time method was utilized to formulate our search strategy. The patient population was defined as adult patients greater than 18 years of age. The intervention was a mini-open ATR in this population. Comparative studies were included if they compared mini-open repair patients to traditional open or percutaneous repair. The outcomes in this study consisted of rates of RTS, RTA, and RTW, rates of rerupture, complications, and quantifiable PROs. Studies with any follow-up period were included. Studies were included if they reported outcomes of mini-open Achilles repair after an Achilles tendon rupture and in patients 18 years or older. Exclusion criteria included case reports, review articles, technique articles, cadaveric studies, expert opinions, articles without outcomes reported, and articles not in English. Two reviewers independently analyzed all articles included in this study, and if they were not unanimous in their decision, a third reviewer was consulted to determine study inclusion or exclusion. Articles underwent further review until a consensus was reached to determine article inclusion. All included studies underwent a thorough reference search to determine whether additional studies could be added to this review. A manual gray literature search was also performed to find additional studies that may have been missed in the original systematic search of the three databases. This protocol is registered in the PROSPERO database.

Quality Assessment

Three authors used the Methodological Index for Nonrandomized Studies (MINORS) criteria to score all articles based on their study quality [9]. MINORS criteria were scored 0 (not reported), 1 (reported but inadequate), or 2 (reported and adequate), with a maximum score of 16 for non-comparative studies and 24 for comparative studies. Each author scored each article individually before reviewing their scores. Any discrepancies in scores were resolved by rereviewing the articles until a consensus was reached. MINORS criteria that scored 1 or 2 for at least seven or more sections (11 or more for comparative studies) were considered low risk of bias, a score of 1 or 2 for five to six sections (9-10 categories for comparative studies) were considered moderate risk of bias, and a score of 1 or 2 for four categories or less (eight or less categories for comparative studies) were considered high risk of bias.

Data Extraction and Statistical Analysis

Study variables analyzed in this systematic review included title, author, publication date, study year, number of patients/Achilles, mean age, mean follow-up time, time to RTA/RTS/RTW, preoperative and postoperative PROs, and rates of complications. All extracted data were analyzed using Google Sheets (Google Drive; Google, Mountain View, CA). Descriptive statistics such as mean, percentage, standard deviations, and ranges are reported when applicable and available.

Results

Literature Selection

**Figure 1:**
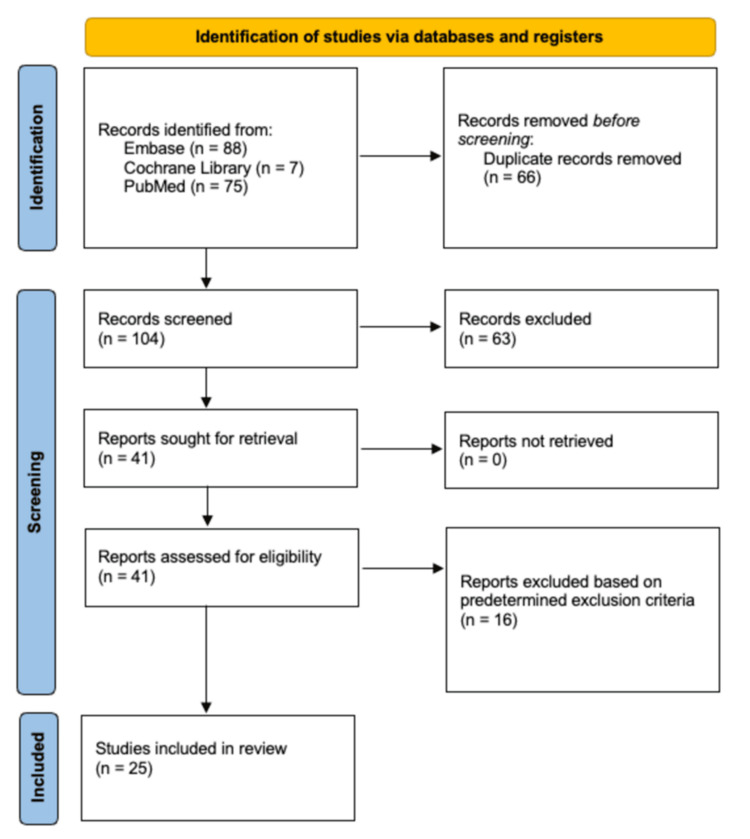
PRISMA flow diagram depicting the article selection process PRISMA: Preferred Reporting Items for Systematic reviews and Meta-Analyses Image credit: This is an original image created by the author Daniel I. Razick

The initial search yielded 170 articles through PubMed, Embase, and Cochrane Library. From there, 66 duplicates were removed, leaving 104 articles. These articles were then reviewed by title and abstract, narrowing them down to 41 articles, excluding 63. A full-text review was then conducted for the remaining 41 articles, yielding 25 total studies to be included in the systematic review, which is shown in Figure 1 [6,10-33].

Patient Characteristics of Included Studies

Of the 25 studies included in this review, 11 evaluated outcomes of mini-open repair alone, 12 compared outcomes of mini-open repair with either open or percutaneous repair, two studies compared two different methods of performing a mini-open repair, and one study evaluated outcomes of acute vs. delayed mini-open repair. There were 957 patients (82.5% male; 17.5% female) with a mean age of 39.34 ± 6.84 years who underwent mini-open repair. The mean time from initial injury to repair was seven days (range, 2.6-16 days). The mean follow-up period was 29.6 months (range, 6-117.6 months). Table 1 summarizes study characteristics and patient demographic information.

**Table 1:** Study characteristics and patient demographics ^*^Median value Age, body mass index, time from injury to repair, and follow-up are reported as mean ± standard deviation (range) when available NR: not reported; LOE: level of evidence

Study	Journal	Study year	LOE	Number of patients (M/F)	Age (years)	Body mass index (kg/m^2^)	Time from injury to repair (days)	Follow-up (months)	
Akoh et al. [10]	Foot & Ankle International	2010-2018	4	33 (26/7)	40^*^ (22-78)	27.9^*^ (19.7-47.6)	10^*^ (1-45)	44.4^*^ (12-117.6)	
Anathallee et al. [11]	Foot and Ankle Surgery	2007-2009	4	Acute: 10 (8/2)	51 (33-73)	NR	5.9 (1-9)	71 (58-92)	
				Delayed: 14 (10/4)	45 (21-73)	NR	16 (11-31)		
Assal et al. [12]	Journal of Bone and Joint Surgery	1996-1999	3	79	36.5 (22.5-82)	NR	NR	26 (18-42)	
Bhattacharyya and Gerber [13]	International Orthopaedics	NR	4	25	42	NR	3.6 (0.5-7)	12	
									Calder and Saxby [14]	British Journal of Sports Medicine	NR	3	46 (31/15)	40 (22-69)	NR	7 (1-42)	12	
Daghino et al. [6]	Injury	2010-2014	4	68 (59/9)	43.1 ± 9.9	25.4 ± 3.2	7 ± 3	Minimum 6 months	
									De Carli et al. [15]	Journal of Sports Medicine and Physical Fitness	1995-2001	4	20 (14/6)	39.7 (38-57)	NR	NR	52 (20-95)	
Drogomiretskiy et al. [16]	Foot & Ankle Surgery: Techniques, Reports, & Cases	2016-2019	3	10 (6/4)	42.8 ± 11.6 (21-56)	30.4 ± 4.9 (21.2-39.6)	NR	23.9 ± 5.1	
								14.7 ± 1.9	
Fu and Qu [17]	The Surgeon	2006-2010	2	30 (22/8)	39.2 (22.8-54)	NR	4	25 (18-48)	
									Hoskins et al. [18]	Foot & Ankle Specialist	2013-2020	3	81 (68/13)	41.4 ± 10.7	27.1 ± 4.2	NR	38.4 (12-71)	
Keller et al. [19]	The American Journal of Sports Medicine	2005-2011	4	100 (91/9)	42 ± 11.9	NR	NR	42.1 ± 22.4										
Klein et al. [20]	Foot & Ankle Specialist	2002-2010	3	18	46 ± 2.5 (33-73)	NR	15 ± 2 (2-30)	Minimum 12 months	
									Ling et al. [22]	The Foot	2008-2010	4	12	41 ± 12.7 (27-62)	NR	NR	Minimum 60 months	
Li et al. [21]	BMC Musculoskeletal Disorders	2016-2018	3	34 (31/3)	32.3 ± 6.9 (21-42)	24.3 ± 2.7	NR	29 ± 2.9	
									McKissack et al. [23]	Cureus	2011-2018	4	26 (24/2)	31.4	27.2	NR	Minimum 6 months	
Mukundan et al. [24]	Foot and Ankle Surgery	2004-2007	4	21 (8/13)	43.4	NR	NR	Minimum 12 months										
Munegato et al. [25]	Muscles, Ligaments, and Tendons Journal	NR	4	17 (14/3)	47.2 ± 11.8	NR	4.7 ± 3	24	
									Nguyen et al. [26]	Annals of Medicine and Surgery	2017-2020	3	21 (17/4)	35.3 (18-55)	NR	4.8 ± 2.8 (2-8)	18 (12-36)	
Park et al. [27]	The American Journal of Sports Medicine	2012-2019	3	Achillon: 20 (15/5)	40.9 ± 10.2	26.2 ± 3.1	2.9 ± 2.2	27.2 ± 12.1	
				Ring forceps: 30 (26/4)	41.2 ± 13.8	25.1 ± 2.6	2.6 ± 1.3	27.3 ± 10.2	
Rebeccato et al. [28]	The Journal of Foot & Ankle Surgery	1992-1997	3	Mini-open: 22	40.3 (16-67)	NR	NR	21	
									Sanada et al. [29]	The Journal of Foot & Ankle Surgery	2014-2015	3	44 (28/16)	41.5 (23-71)	NR	NR	8.5 (6-15)	
																		Sharaby et al. [30]	Techniques in Foot and Ankle Surgery	2017-2018	1	(Modified) mini-open: 20 (18/2)	33.5 (19-47)	NR	NR	27.1 (12-38)	
(Conventional) mini-open: 20 (19/1)	31.8 (27-39)																									
Taşatan et al. [31]	The Journal of Foot & Ankle Surgery	2003-2008	3	20 (18/2)	39.3 (21-55)	NR	NR	58.5 (18-60)	
Vadalà et al. [32]	Journal of Sports Medicine and Physical Fitness	2000-2010	3	80 (62/18)	33.9 ± 8.2	NR	NR	58 (26-116)	
Vadalà et al. [33]	Muscles, Ligaments, and Tendons Journal	2008-2010	3	36 (33/3)	29.7 (21-35)	NR	NR	28 (24-31)	

Methodological Quality and Risk of Bias

Thirteen studies had a score between 9 and 14, eight studies scored between 15 and 19, and four studies scored between 20 and 24. The risk of bias was determined to be low in five, moderate in 14, and high in five studies. MINORS scores for all included studies are summarized in Table 2.

**Table 2:** Methodological quality and risk of bias

Study	Clearly stated aim	Inclusion of consecutive patients	Prospective data collection	Endpoints appropriate to study aim	Unbiased assessment of study endpoint	Follow-up period appropriate to study aim	Loss to follow-up less than 5%	Prospective calculation of study size	Adequate control group	Contemporary groups	Baseline equivalence of groups	Adequate statistical analyses	Total score
Akoh et al. [10]	2	2	2	2	0	2	2	0	-	-	-	-	12/16
Anathallee et al. [11]	2	2	2	2	0	2	1	0	1	2	2	0	16/24
Assal et al. [12]	2	2	2	2	0	2	2	1	-	-	-	-	13/16
Bhattacharyya and Gerber [13]	2	2	2	2	0	1	2	0	2	2	2	0	17/24
Calder and Saxby [14]	2	1	2	2	0	2	2	0	-	-	-	-	11/16
Daghino et al. [6]	2	2	2	2	0	2	2	1	2	2	2	1	20/24
De Carli et al. [15]	2	1	2	2	0	2	2	0	-	-	-	-	11/16
Drogomiretskiy et al. [16]	2	2	2	2	0	2	1	1	0	2	2	1	17/24
Fu and Qu [17]	2	2	2	2	1	2	2	1	2	2	2	1	21/24
Hoskins et al. [18]	2	2	1	2	0	2	0	0	2	2	0	2	15/24
Keller et al. [19]	2	2	0	2	0	2	1	2	-	-	-	-	11/16
Klein et al. [20]	2	2	1	2	0	2	1	0	2	2	1	2	17/24
Ling et al. [22]	2	2	0	2	0	2	0	0	0	0	1	2	11/24
Li et al. [21]	2	2	2	2	0	2	1	2	2	2	2	2	21/24
McKissack et al. [23]	2	1	0	2	0	2	0	0	1	2	1	2	13/24
Mukundan et al. [24]	2	0	1	2	0	2	1	0	1	2	2	1	14/24
Munegato et al. [25]	2	0	0	2	1	2	1	0	2	2	2	2	16/24
Nguyen et al. [26]	2	2	2	2	0	1	0	0	-	-	-	-	9/16
Park et al. [27]	2	2	2	2	0	2	2	0	0	1	0	2	15/24
Rebeccato et al. [28]	2	2	1	2	0	1	2	0	0	2	1	0	13/24
Sanada et al. [29]	2	2	2	2	1	1	2	0	1	2	2	2	19/24
Sharaby et al. [30]	2	2	2	2	1	1	2	0	2	2	2	2	20/24
Taşatan et al. [31]	2	2	2	2	0	2	2	0	-	-	-	-	12/16
Vadalà et al. [32]	2	2	2	2	0	2	0	0	-	-	-	-	10/16
Vadalà et al. [33]	2	2	2	2	0	2	2	0	-	-	-	-	12/16

RTA, RTW, and RTS

The overall rate of RTA across eight studies (235 patients) was 96.46% (82.3%-100%), ranging from a mean time of four months (3.8-7). Five of the eight studies (172 patients) reported 100% RTA at preinjury levels. Nguyen et al. reported RTA for light activities only.

The overall rate of RTS across 12 studies (571 patients) was 92.7% (82.3%-100%) with a mean time of 5.4 (4.3-6.1) months. Ten studies had 414 (72.5%) patients who returned to their preinjury sports levels. In terms of the level of sport, Assal et al. included five high-level athletes who were members of the Swiss national team. Vadalà et al. had seven professional players and 60 amateur players. The other 10 studies did not report on the level of sport.

Keller et al. reported that 80 patients (80%) returned to their previous or higher levels of sport, five patients (5%) returned to a lower level, eight patients (8%) changed sports, and seven patients (7%) did not return to sport. Mukundan et al. reported 20 patients (95%) with RTS, with one patient (5%) not returning due to fear of rerupture despite reassurance. Sharaby et al. reported a 100% rate of RTS; however, only one patient (5%) returned at preinjury levels, whereas 17 patients (85%) returned with minimal restriction and two patients (10%) with moderate restriction. Vadalà et al. reported that of 80 total patients, 67 (83.75%) had RTS at preinjury levels, whereas 13 (16.25%) did not resume due to fear or residual pain. The overall rate of RTW across eight studies (308 patients) was 100%, ranging from a mean time of 2.2 (0.6-4.5) months.

Postoperative PROs

The mean postoperative American Orthopedic Foot and Ankle Score (AOFAS) was 97.1 (90.1-99.2) across 11 studies with 494 patients. The mean postoperative Achilles Tendon Total Rupture Score (ATRS) was 90.3 (86-94.6) across eight studies with 301 patients. The mean postoperative Visual Analogue Scale (VAS) Pain score was 2.64 (0.2-8.85) across four studies with 91 patients. Surgical outcomes, PROs, RTA/RTS/RTW, and complications are summarized in Table 3.

**Table 3:** Surgical outcomes and RTW/RTA/RTS ^*^95% Confidence interval PRO: patient-reported outcome; RTW: return to work; RTA: return to activity; RTS: return to sport; FADI: Foot and Ankle Disability Index; FAOS: Foot and Ankle Outcome Score; ADL: activities of daily living; QOL: quality of life; VAS: visual analogue scale; NR: not reported; ATRS: Achilles Tendon Total Rupture Score; AOFAS: American Orthopedic Foot and Ankle Score; MFS: Maryland Foot Score; SEFAS: Self-Reported Foot and Ankle Score; DVT: deep vein thrombosis; VISA-A: Victorian Institute of Sport Assessment-Achilles

Study	Number of patients (n)	Mean follow-up (months)	Postoperative PROs	Complications	Reoperations	Return to work/activity/sport			
						RTW	RTA	RTS	
Akoh et al. [10]	33	44.4	FADI: 98.3 (96.6-100)^*^; FAOS pain: 99.2 (97.7-100.7)^*^; FAOS symptoms: 97 (95.3-98.7)^*^; FAOS ADL: 97.2 (91.6-102.7)^*^; FAOS sports: 98.5 (96.3-100.6)^*^; FAOS QOL: 88.7 (82.7-94.7)^*^; VAS: 0.2 (0.1-0.4)^*^	None	None	NR	100% at preinjury level at 5.6 (1.7-22.1) months	NR	
Anathallee et al. [11] (acute)	10	71	ATRS: 94.6 (85-100); Leppilahti: 88 (65-100)	Sural nerve lesion (2)	None	NR	NR	NR	
Anathallee et al. [11] (delayed)	14	71	ATRS: 91.3 (55-100); Leppilahti: 81.4 (30-100)	Rerupture (2); sural nerve lesion (5); wound problem due to sinus development from undissolved suture (1)	Revision (1)	NR	NR	NR	
Assal et al. [12]	79	26	AOFAS: 96 (85-100)	Reruptures (3) (2 due to noncompliance and 1 due to a fall at 12 weeks)	Open revision (3)	100%	NR	100% (5 were Swiss national athletes)	
Bhattacharyya and Gerber [13]	25	12	NR	None	None	NR	100% at 3.25 (3-5.3) months	NR	
									Calder and Saxby [14]	46	12	AOFAS at 3 months: 95.8 (85-100); AOFAS at 6 months: 98.4 (95-100)	Superficial infection (1); sural nerve lesion (2)	None	22 (4-77) days	NR	100% at preinjury level by 6 months	
Daghino et al. [6]	68	6	NR	Rerupture (2)	None	NR	NR	87.5% by 6.1 ± 2.5 months; 73.5% at preinjury level	
									De Carli et al. [15]	20	52	VAS pain: 8.85 (5-10)	Mild pain after prolonged weight-bearing (2); mild pain after normal weight-bearing (1)	None	<3 months in 85% and >3 months in 15%	NR	85% by 5 months (3-8); 70.5% at preinjury level; 74.6% resumed the same sport activity	
Drogomiretskiy et al. [16]	10	23.9	MFS: 95.7 ± 9; SEFAS: 43 ± 4.7; Satisfaction: excellent (9), good (0), fair (1), failure (0)	None	None	NR	100% at preinjury level	NR	
									Fu and Qu [17]	30	25	AOFAS: 97.3 ± 4; ATRS: 86 ± 5.4	Delayed wound healing (1)	None	NR	100% at preinjury level	NR	
Hoskins et al. [18]	81	38.4	AOFAS: 90.3; ATRS: 86.96	Rerupture (1) (due to noncompliance); superficial skin infection (1)	Revision (1)	NR	100% at preinjury level	NR										
									Keller et al. [19]	100	42.1	AOFAS: 97.7 ± 5; Leppilahti: 54.2 (35-63)	DVT (5); local discomfort (2); rerupture (2)	NR	100% by 56 ± 15.4 days	NR	80% at preinjury level at 18.9 ± 4.4 weeks; 5% at lower level; 8% changed sport; 7% did not RTS	
Klein et al. [20]	18	12	VISA-A mini: 92 ± 5 (66-100)	Partial rerupture (1); wound dehiscence (1)	Revision (1)	NR	100% at preinjury level by 5 ± 0.6 (4-11) months	NR	
									Ling et al. [22]	12	60	VAS: 0.5 ± 1.2 (0-3); AOFAS: 97.42 ± 2.4 (91-100); FAOS symptoms: 87.5 ± 16.3 (54-100); FAOS pain: 100; FAOS ADL: 96.83 ± 4.7 (87-100); FAOS sports: 92.08 ± 7.5 (70-100); FAOS QOL: 88.17 ± 14.5 (50-100)	Stitch abscess (1)	None	NR	NR	NR	
Li et al. [21]	34	29	AOFAS: 95.0 ± 3.8; ATRS: 93.8 ± 3.8	None	None	NR	NR	NR	
									McKissack et al. [23]	26	6	VAS pain: 1	Superficial wound infection (1), wound dehiscence (4)	Revision (1)	NR	NR	NR	
Mukundan et al. [24]	21	12	AOFAS: 95.14 ± 3.18; Leppilahti: 95.24 ± 3.93	Superficial wound infection (1)	None	NR	NR	95% at preinjury level in 6 months										
Munegato et al. [25]	17	24	ATRS: 92.71 ± 13 (55-100); modified Leppilahti: 47% (8/17) excellent, 35% (6/17) good, and 18% (3/17) fair	Late heel ulcer secondary to boot wear (1)	None	NR	NR	82.3%	
									Nguyen et al. [26]	21	18	AOFAS: 97.2 ± 1.6; ATRS: 91.2 ± 1.8	None	None	100% at 19.7 ± 0.9 (18-22) days	100% at 16.9 ± 1.1 (15-18) weeks	NR	
Park et al. [27] (Achillon mini-open)	20	27.2	AOFAS: 90.1 ± 8.7; ATRS: 88.3 ± 9.9	Rerupture (1); superficial wound infection (1)	Open revision (1)	NR	NR	NR	
Park et al. [27] (forceps mini-open)	30	27.3	AOFAS: 92.2 ± 9.4; ATRS: 89.9 ± 10.9	None	None	NR	NR	NR	
Rebeccato et al. [28]	22	21	NR	Rerupture (1), delayed wound healing (1)	Open revision (1)	100% at 19 (0-90) days	NR	NR	
									Sanada et al. [29]	44	8.5	ATRS: 88.4	Reinjury (1)	None	NR	NR	100% at 24.8 weeks	
																		Sharaby et al. [30] (modified mini-open)	20	27.1	Weber modification of Thermann score: 86.2 (78-96)	Surgical site tenderness with scar thickening (1)	None	NR	NR	5% at preinjury level; 85% with minimal restriction; 10% with moderate restriction	
Sharaby et al. [30] (conventional mini-open)	20	27.1	Weber modification of Thermann score: 83.2 (75-90)	Sural nerve lesion (7); surgical site tenderness with scar thickening (3); superficial wound thickening (2)	None	NR	NR	15% at preinjury level; 75% with minimal restriction; 10% with moderate restriction	
Taşatan et al. [31]	20	58.5	AOFAS: 99.2; Trillat score: 19 very well, 1 well	None	None	100%	NR	100% at preinjury level	
Vadalà et al. [32]	80	58	VISA-A: 63 (78.75%) excellent, 14 (17.5%) good score, 2 (2.5%) fair, 1 (1.25%) poor Hannover, 63 (78.75%) excellent, and 17 (21.25%) good	Hypertrophic scar (1); late skin adhesions (9); wound healing delay (2)	None	NR	NR	83.8% at preinjury level; 16.2% did not resume due to fear or residual pain	
Vadalà et al. [33]	36	28	VISA-A: 24 (66.6%) excellent, 11 (30.5%) good, 1 (2.7%) poor; Hannover: 27 (75%) very good, 9 (25%) good	Skin adhesions (2); hypertrophic scar (1); delayed wound healing with superficial infection (3)	None	NR	NR	100% with 91.7% at preinjury level at 5-10 months	

Patient Complications

In total, 77 (8.05%) complications were reported in the mini-open patients across all studies. Reported complications included 16 sural nerve injuries (1.67%), 12 reruptures (1.25%), 17 skin adhesions (1.77%), five infections (0.5%), eight delayed wound healing (0.84%), five wound dehiscence (0.5%), and five deep vein thromboses (0.52%). Other complications included mild pain after weight bearing, local discomfort, stitch abscess, late heel ulcer, surgical site tenderness, hypertrophic scar, and superficial wound thickening. It is noteworthy to indicate that six studies with 143 (14.94%) patients reported no complications.

Li et al. compared mini-open with percutaneous repair and found that the latter group had a significantly greater incidence of sural nerve injury (0% vs. 14.7%; p = 0.027). Sharaby et al. evaluated a traditional mini-open approach to their modified technique and found a significant reduction in sural nerve injury for the modified approach (35% vs. 0%, p = 0.003). In an open and mini-open comparison by Daghino et al., there was a significant reduction (p < 0.001) in the occurrence of minor complications, primarily due to decreased delayed wound healing and scar adhesion incidence in the mini-open group (p < 0.05). Similarly, Fu and Qu found a significant decrease in complications in the mini-open group compared to the open (3.33% vs. 26.7%, p = 0.01).

Discussion

This systematic review evaluated 25 clinical studies intending to assess the outcomes of patients undergoing mini-open ATR by analyzing PROs, complication rates, and rates of RTA/RTS/RTW. The main findings in this systematic review were that mini-open ATR techniques demonstrated the following: 1) high rates of RTS/RTW/RTA levels ranging from 82.3% to 100%, 2) high postoperative PRO scores, and 3) low rates of complications. The mini-open technique combines the properties of open and percutaneous techniques to reduce operative complications while maintaining the strength of the repair achieved in open procedures [34]. A small longitudinal incision allows for direct visualization for accurate suturing, which allows for proper tendon alignment [35]. The choice between operative techniques should consider the biomechanical properties of the ankle joint. After repair, there is a noticeable decrease in the total range of motion of the affected side [36]. The decrease in range of motion can be attributed to prolonged periods of immobilization, causing tendon adhesions. Additionally, the ends of the tendon are debrided, approximated, and sutured. The tendon subsequently shortens [36]. Mini-open repair preserves the paratenon, which provides vascularity, facilitating Achilles tendon healing [37]. The minimally invasive nature of this procedure maintains a better vascular supply and can result in faster tissue remodeling to facilitate earlier return to activities [37].

Functional Outcomes

The included studies reported significant heterogeneity in functional outcomes and postoperative PROs. Additionally, most of the included studies did not include preoperative PROs to compare with the postoperative PROs. However, 23 (92%) studies included at least one postoperative PRO.

Twenty (80%) studies reported RTA, RTW, or RTS. Eight (32%) studies reported on RTA, with a mean rate of 96.46% (82.3%-100%), and a mean time of 4 (3.8-7) months. The results in this systematic review are similar to a study by Hsu et al., which found that 88% of patients were able to return to baseline activity by five months postoperatively, with 98% of those being patients who were treated with a mini-open technique (p = 0.0001) [38]. Eight (32%) studies reported on RTW, with all patients returning to work, at a mean of 2.2 (0.6-4.5) months. McMahon et al. found that patients undergoing MIS returned to work on average by the sixth postoperative week compared to the ninth with open repair [39]. Calder and Saxby found that the average RTW was 22 days [14]. Twelve (48%) studies reported on RTS, with a mean rate of 92.7% (82.3%-100%) and a mean time of 5.4 (4.3-6.1) months. These findings are similar to a 2015 systematic review that also found that patients returned to sport within six months after a mini-open repair [40]. These results are also similar to a 2017 systematic review and meta-analysis of return to play after an Achilles tendon rupture, which found an average return to play at six months [41]. The earlier recovery in patients treated with mini-open procedures may be attributed to the smaller incision and subsequently reduced likelihood of wound-healing issues. Among athletes, the main benefit of ATR repair is not just the return to daily activities but also the restoration of the triceps surae strength and endurance, which are essential for sports activities.

Postoperative PROs, such as mean AOFAS score of 97.1 (90.1-99.2), ATRS score of 90.3 (86-94.6), and VAS score of 3.35 (0.2-8.85), had excellent results after mini-open repair. These findings are similar to a recent 2023 systematic review and meta-analysis from Attia et al. evaluating minimally invasive repair vs. open repair that found a statistically significant improvement in ATRS scores but not for AOFAS [8]. However, Grassi et al. found a statistically significant improvement in AOFAS [7]. The higher PRO scores can be in part due to the reduced number of suture knots required with mini-open repair compared to percutaneous repair. This causes a decreased sensation of foreign bodies and keloid formation, which improves function and cosmesis of the ankle [36]. Additionally, through a biomechanical cadaveric study, the mini-open/open approach reduced the tendon's early elongation compared to the percutaneous approach [42]. This suggests a more robust repair, but the ultimate strength of the repair assessed through cycles to failures was comparable across the open/mini-open/percutaneous techniques in the study [43]. However, the reduced early elongation of repair associated with open/mini-open may allow earlier initiation of postoperative rehabilitation and subsequently earlier return to activities.

Complications

Overall, the complication rate for mini-open repair in this study was 8.05%. Hsu et al. found no significant difference in complications between the Percutaneous Achilles Repair System (5%) and open repair (10.6%), including rerupture (p = 1.0), sural nerve injury (p = 0.16), superficial and/or deep infection (p = 0.29 for both), wound dehiscence (p = 0.74), and reoperation (p = 0.13) [39]. Several previous systematic reviews and meta-analyses have found that minimally invasive techniques, such as mini-open, have similar rerupture rates to open repairs [7,8,35,40,43].

Historically, percutaneous repair had a high rate of sural nerve injury, up to 60%, as reported decades ago by Ma and Griffith [5]. Sural nerve injury remains one of the most common complications from minimally invasive repair. The procedure involves making several small incisions and blind suturing, which has a higher risk of sural nerve injury as well as rerupture due to nonanatomical tendon contact [36]. The blind passage of needles and suturing can lead to variable fixation, which can cause gapping or earlier elongation under load. However, as new techniques emerge and alterations to current techniques evolve (e.g., ultrasound guidance, lateral incisions, and new suture patterns), the repair strength has improved, and rates of complications have decreased [36]. A meta-analysis of mini-open vs. percutaneous repair found a significantly lower rerupture rate (1.48% vs. 6.11%) and sural nerve injury (0.54% vs. 5.67%) [36]. Gapping at the repair site decreases healing and predisposes the tendon to rerupture [44]. Thus, through direct visualization, mini-open repair can ensure no gap between the approximated tendons, leading to a strong repair.

The high rates of RTA/RTW/RTS and low complication rates make the mini-open approach an attractive option for patients who wish for a quicker return and for older patients who may have lower functional outcomes, prolonged recovery periods, and higher rates of complications [45]. Future studies can focus on evaluating these outcomes in different age groups undergoing mini-open repair. More high-quality trials comparing mini-open vs. other surgical modalities with standardized methodologies are still required to provide deeper insight into the outcomes of mini-open repair.

Limitations

This systematic review demonstrates several limitations. First, there is heterogeneity and considerable risk of bias in the included studies. Differences in rehabilitation protocols are a major confounding factor that can impact the results. Early range of motion and initiation/timing of rehabilitation can impact the functional outcomes seen. Second, the majority of studies did not report any preoperative PROs. Therefore, this prevents us from comparing the improvement and significance of the postoperative PROs. This heterogeneity and limited preoperative PROs preclude a meta-analysis being done. Third, heterogeneity regarding operative technique, PROs, and clinical outcomes was reported. Fourth, RTA/RTW/RTS is self-reported and can vary in meaning between patients. Fifth, levels of activity varied between studies, which may impact the rates of return. Sixth, publication bias, in which studies that have demonstrated favorable outcomes as opposed to negative outcomes, may have impacted the overall results of this study.

## Conclusions

Mini-open repair demonstrated an early return to baseline activities, high PRO scores, and low rates of complications/reruptures. These results demonstrate that mini-open provides satisfactory functional outcomes for Achilles tendon rupture. However, given the limitations of the present systematic review, the inability to perform a meta-analysis given the significant heterogeneity of outcomes presented, and possible publication bias, no definitive conclusion can be made at this time. This study highlights the need for future direct comparisons of mini-open repair with conventional open repair of the Achilles tendon through randomized controlled trials.
